# Rocky Mountain spotted fever contracted along a Canadian road trip: A case report

**DOI:** 10.1177/2050313X241260980

**Published:** 2024-06-14

**Authors:** Aliyah King, Alison Spurr, Reetesh Bose

**Affiliations:** 1 Faculty of Medicine, University of Ottawa, Ottawa, ON, Canada; 2 Division of Dermatology, University of Ottawa, and The Ottawa Hospital, Ottawa, ON, Canada

**Keywords:** Rocky Mountain spotted fever, tick-borne disease, case report

## Abstract

Rocky Mountain spotted fever, a potentially fatal tick-borne disease thought to be confined to specific climates and geographic locations, is expanding its reach due to climate change. This is demonstrated by a 73-year-old woman who contracted Rocky Mountain spotted fever outside endemic areas during travel in Canada. Presenting with fevers, arthralgia, weakness, non-bloody diarrhea, conjunctivitis, mild cough, and a rash, this patient was initially started on moxifloxacin (400 mg PO/day) for suspected pneumonia. Treatment was changed to doxycycline (100 mg PO twice daily for 7 days) after dermatology was consulted, and Rocky Mountain spotted fever was thought to be higher on the differential. Rocky Mountain spotted fever was confirmed, and the patient responded well to antibiotics, improving by discharge. The disease’s expansion into previously thought nonendemic areas is thought to be linked to milder winters and more extreme dry summers, facilitating pathogen development and tick lineage expansion.

## Introduction

Rocky Mountain spotted fever (RMSF) is a tick-borne systemic, small-vessel vasculitis of the bacteria *Rickettsia rickettsii*, primarily transmitted by the vectors *Dermacentor variabilis* (American dog tick), *Dermacentor andersoni* (Rocky Mountain wood tick), and *Rhicephalus sanguineus* (Brown dog tick).^1,2^ Cutaneous manifestations include an initial erythematous rash, which can progress into a morbilliform or petechial eruption, usually starting on the extremities and spreading to the trunk and head.^1,2^ The incubation period is 2–14 days, with a classic triad of fever, rash, and headache.^2^ The mortality rate is 25% without effective therapy, which is typically using oral doxycycline.^2,3^ RMSF incidence is highest during warm and dry seasons, with over 90% of cases reported between April and September.^1,2^

RMSF reporting varies and is limited across provinces and territories. In Canada, RMSF is primarily found in British Columbia, with an incidence of 0.20 per 100,000 population in 2019.^4^ Specifically, *D. andersoni* is most common in the dry areas of British Columbia, Southern Alberta, and Southern Saskatchewan, whereas *D. variabilis* spans Eastern Saskatchewan to Nova Scotia.^5^ A multicriteria decision analysis ranked RMSF as the fourth-highest priority non-endemic disease in Canada and the most concerning for spreading under the current climate conditions.^6^ Prompt diagnosis is crucial as patients may experience seizures, coma, shock, arrhythmias, or myocarditis.^2^ We present the case of a patient who contracted RMSF during a road trip from Saskatchewan to Ontario.

## Case

**Figure 1.**
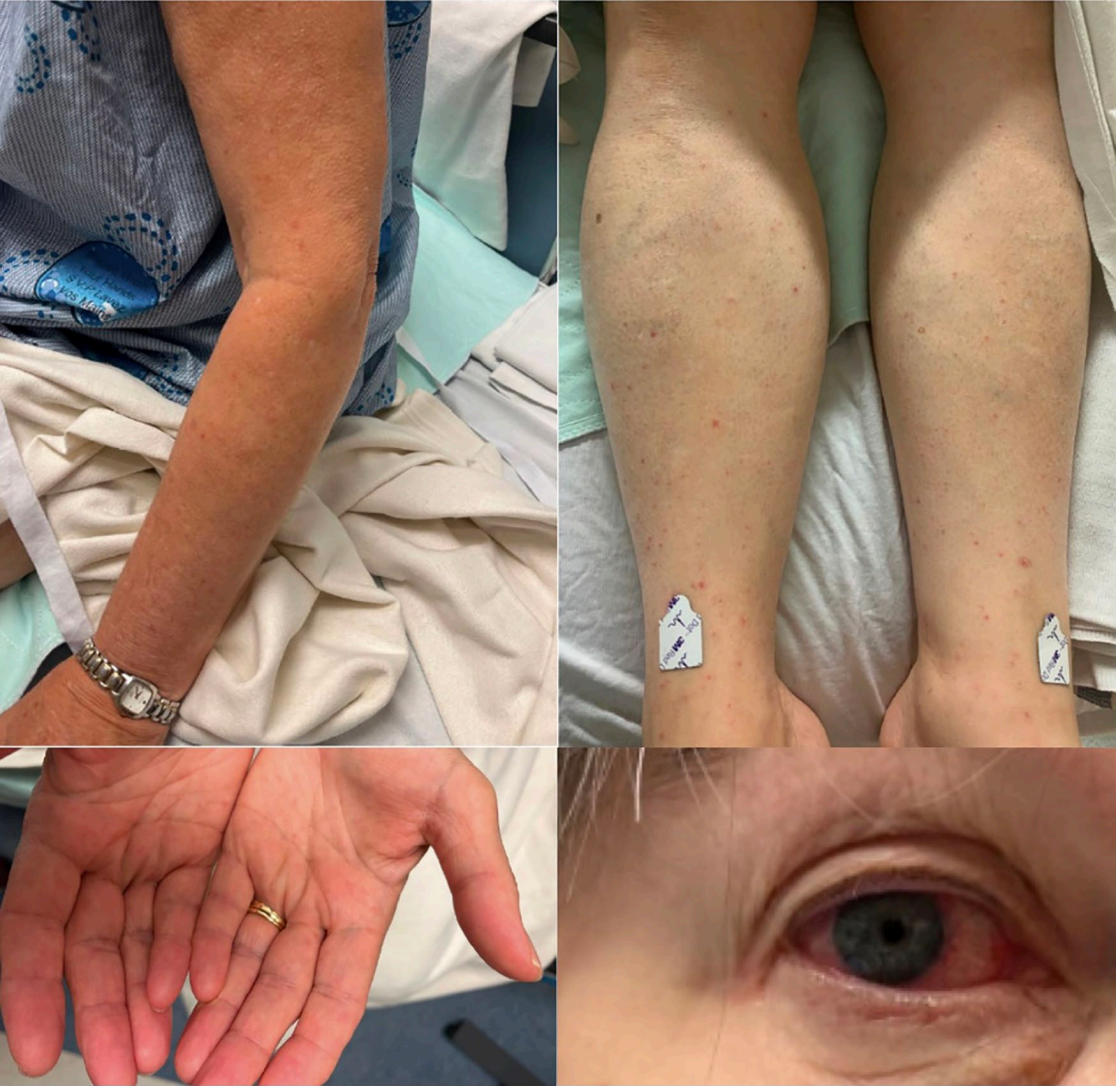
Unilateral conjunctivitis and discrete, petechiae and palpable purpuric papules on patient’s arm, legs, and palmar surfaces.

A 73-year-old female presented to the emergency department in mid-July 2023, with a 1-week history of sudden-onset fevers (elevated C-reactive protein of 118 and erythrocyte sedimentation rate of 30), arthralgia, progressive weakness, 3–4 watery bowel movements per day, conjunctivitis (Figure 1), and a mild dry cough. Cutaneous presentation included discrete, 1–2 mm non-blanchable petechia, and palpable purpuric papules on her arms, legs, and palmar surfaces (Figure 1). The rash started on her legs and hands, then spread to her torso and arms, with no associated necrotic eschar. The patient appeared generally well, with a blood pressure of 100/62, a heart rate of 70, and a temperature of 36.4°C. There was no cervical or head lymphadenopathy and no clinical signs of meningitis such as photophobia or neck stiffness. A physical examination revealed normal heart sounds with no murmurs, good air entry in both lungs, a soft and non-distended abdomen that was non-tender, and no focal neurological deficits other than an episode of diplopia in the emergency department that was self-limited.

The patient had a medical history of non-alcoholic steatohepatitis, and no regular medications or allergies. There was no history of autoimmune diseases or previous skin conditions. The patient lives with her husband in Saskatchewan, is a non-smoker, and non-drinker, and does not use recreational drugs. Her last international travel was in September 2022 to Portugal; however, she had traveled to Quebec City, Quebec, and Ottawa, Ontario by train 1 week before symptom onset. There were no instances of camping, exposure to wooded areas, tick or insect bites, or swimming in lakes. All travel in Quebec and Ontario had been within major cities. The patient had no known sick contacts, and the only animal exposure was her son’s two dogs when she stayed in Ottawa. She had gone on a cruise on the Ottawa River while in Ottawa.

The patient was admitted for investigation and dermatology was consulted. Community-acquired pneumonia had been suspected and she was started on moxifloxacin (400 mg per oral daily from July 15 to 17, 2023). The remainder of the baseline infectious and vasculitis workup was unremarkable.

After assessment by Dermatology, RMSF and other rickettsial illnesses were strongly considered given her prodromal illness and rash. They were started on empiric treatment with doxycycline 100 mg oral, twice a day for 7 days. Additional differential diagnoses considered were post-viral eruptions, pityriasis lichenoides et varioliformis acuta, secondary syphilis, vasculitis, and autoimmune connective tissue disease. The work-up returned with positive serology for RMSF (titer 1:2048) and the remainder of testing negative for syphilis, legionella, leptospirosis, and other tick-borne illnesses. The patient made a full recovery and was discharged back to their home.

## Discussion

This is a case of a 73-year-old female who contracted RMSF during her road trip from Saskatchewan to Ontario expands the understanding of the disease’s geographical distribution and its association with climate change. Previously, climate features were believed to restrict the expansion of tick lineages carrying RMSF.^7^ Tropical lineages were thought to be confined to 20–30°C and temperate lineages to areas north and south of these temperatures.^7^ However, in 2016, tropical lineages of *R. sanguineus* were found in areas with annual average temperatures of 17.6–18.8°C (i.e., San Diego and Lytle Creek).^7^ This may be attributed to climate change, causing milder winters and more extreme dry summers.^8^ Warmer temperatures could facilitate pathogen development in ticks, and increased food availability and reduced winter die-offs could boost animal reservoir populations.^9^ Ecological niche modeling predicts the expansion of *D. andersoni* into Northern Canada.^8^ Given the seriousness of RMSF, physicians should be aware of the possibility of disease presentation in previously nonendemic areas, as seen in this case.^10^
